# Biodegradable temporizing matrix in the management of exposed cortical bone without periosteum: a flap-sparing and reproducible strategy

**DOI:** 10.1016/j.jpra.2026.09.031

**Published:** 2026-09-29

**Authors:** Benjamin Tobalem, Lucas Ungerer, Lauren Ferrero, Jad Bissar, Salomon Bouaziz, Marc Chaouat, David Boccara, Kevin Serror

**Affiliations:** a Université Paris Cité, Faculté de Santé, Paris, France; b Department of Plastic and Burns Surgery, Hôpital Saint-Louis APHP, 1 av. Claude Vellefaux, Paris, 75010, France

**Keywords:** Exposed cortical bone without periosteum, Synthetic dermal matrix, Biodegradable temporizing matrix, Complex wound reconstruction, Flap-sparing strategy, Novosorb® BTM

## Abstract

**Background:**

Exposure of cortical bone without periosteum (CBWP) represent a complex wound with poor healing potential, regardless of its location. Coverage traditionally relies on flaps, a complex reconstruction procedure. The use of a synthetic biodegradable dermal matrix (Novosorb® BTM) may promote formation of a vascularized neodermis over cortical bone, allowing subsequent skin grafting or secondary healing. This study aims to evaluate the effectiveness and limitations of synthetic dermal matrix in covering CBWP, potentially redefining flap indications.

**Methods:**

We conducted a retrospective single-center study including consecutive patients presenting CBWP exposure, regardless of etiology or anatomical site, over a one-year period. Management followed a standardized staged protocol: Surgical debridement ± cortical bone preparation, secure application of Novosorb®, delamination upon homogeneous neodermis formation, followed by split-thickness skin grafting (STSG) or secondary epithelialization. Primary outcomes were complete epithelialization and time-to-event.

**Results:**

19 patients with 22 defects (scalp n = 14, face n = 2, tibia n = 5, chest wall n = 1) were treated with this protocol. Complete healing was achieved in 17 cases (10 by secondary epithelialization, 7 by STSG). Median time to grafting readiness was 37.5 days, and median time to complete healing was 65 days. We identified major complication rate in 4 (flap conversion in 2), and minor complications in 2 cases. At 6 months, all successfully treated wounds remained stable with satisfying functional and aesthetic outcomes.

**Conclusions:**

A staged synthetic dermal matrix (Novosorb® BTM) protocol reliably converts CBWP into a vascularized wound bed suitable for STSG or secondary epithelialization, by promoting neovascularization and granulation tissue formation. It offers temporal predictability and reduces the need for flap reconstruction in selected complex cases.

**Level of evidence:**

IV.

**Lay summary**

Wounds exposing bone without its protective periosteum layer are difficult to treat because the bone surface has a very limited blood supply and does not heal easily. Traditionally, these wounds are covered using complex reconstructive procedures such as local or free flaps, which require longer surgery and may not always be suitable for elderly or fragile patients.

In this study, we evaluated the use of a synthetic dermal matrix called NovoSorb® BTM to help these wounds heal. The matrix acts as a temporary scaffold that allows blood vessels and new tissue to grow over the exposed bone. Once this new tissue layer forms, the wound can be closed with a skin graft or allowed to heal naturally.

Our results show that this staged approach can successfully heal most wounds without the need for flap surgery. This technique may offer a simpler and less invasive treatment option for selected complex wounds.

## Introduction

Soft-tissue defects exposing cortical bone without periosteum (CBWP) are challenging to manage. Spontaneous granulation over bare bone, relatively avascular, is poor, prolonged and unpredictable, precluding primary healing and split-thickness skin grafting (STSG) without first creating a vascularized bed.^1^^,^^2^

This scenario is frequently encountered following wide oncologic scalp resections, where periosteal excision is necessary to ensure oncologic margins.^2^, ^3^, ^4^, ^5^ Similar challenges arise in limb trauma, burns or chronic wounds.

Standard management strategies include prolonged and unpredictable granulation sometimes using negative pressure wound therapy (NPWT), cortical burring and/or fenestration to elicit bleeding and stimulate granulation or flap reconstruction.^6^, ^7^, ^8^ Local, regional, and free flaps are reliable but resource-intensive and carry risks that may be inappropriate in frail oncology or trauma patients.^9^, ^10^, ^11^ A simple, standardized low-morbidity strategy for this common problem remains elusive.^12^

Dermal matrices have emerged over the past decades.^13^, ^14^, ^15^ Acting as a temporary bridge over avascular surfaces, they support fibrovascular ingrowth and neodermis formation beneath a sealing membrane prior to grafting.^1^ Chen was among the first ones in 2010 to present its series of lower limb defects with bone exposure using a staged protocol with dermal matrix and skin graft.^16^

A relatively new material, Novosorb® BTM (Polynovo Biomaterials Pty Ltd, Port Melbourne, Victoria, Australia), a fully synthetic bilayer biodegradable polyurethane matrix, has demonstrated safety and effectiveness across a range of complex wounds, irrespective of etiologies and anatomical site.^17^, ^18^, ^19^, ^20^ Systematic reviews report integration rates exceeding 80% and acceptable infection profiles even in compromised beds.^21^^,^^22^

To our knowledge, no studies have yet specifically evaluated the use of Novosorb® BTM over CBWP as a specific reconstructive entity. Most available evidence is site-specific or derived from heterogenous cohorts.

Standardized studies reporting healing and time-to-event outcomes, across multiple anatomic sites, specifically to explore healing process over CBWP, are lacking.

We hypothesized that a staged synthetic dermal matrix protocol may rapidly, reliably and safely convert avascular CBWP into a graftable bed, regardless of location. The aim of this study was to evaluate outcomes, healing timelines, and complications of our protocol.

## Methods

### Study design and setting

We conducted a retrospective, single-center case series, in our Department of Plastic and Reconstructive Surgery, including patients from November 2024 to October 2025.

### Patients

All consecutive adult patients presenting with soft-tissue defects exposing CBWP with no possibility of direct closure were included, irrespective of anatomic location or etiology. CBWP was defined as surgical removal of the periosteum during oncologic resection or chronically denuded bone appearing as a dry, non-bleeding cortical surface.

Exclusion criteria were the presence of residual periosteum, uncontrolled deep infection, exposed hardware or fracture site, and inability to comply with follow-up.

The modified defect size was defined from a rationale hypothesis and calculated as the maximal linear distance to go through between wound opposing edges to achieve complete closure assuming uniform centripetal granulation from the wound margins.

### Surgical protocol

Surgical management followed a uniform staged protocol.

It began with either oncologic resection (when removal of the periosteum occurred simultaneously) or sharp debridement of the wound including devitalized periosteum. In some oncologic cases, patients required re-excision for positive margins, necessitating additional periosteal removal. For chronically exposed bone, superficial burring and/or drilling was performed under general anesthesia until punctate bleeding was observed.

Novosorb® BTM was then trimmed to fit the defect, applied with the sealing (plastic) membrane facing upward, and fixed tight in intimate contact with the underlying bed using skin staples and/or interrupted sutures circumferentially to eliminate dead space.

The initial dressing consisted in a single layer of non-adherent interface (Jelonet ® or equivalent) directly over the sealing membrane, followed by sterile gauze with aqueous chlorhexidine solution. When required by anatomic site, immobilization was provided.

The first dressing change was performed on postoperative day 2, then every 2 days during the first week, and subsequently twice weekly. The protocol included gentle cleansing with water and mild soap, evacuation of serous collections by light pressure around edges and fenestrations, application of chlorhexidine and reapplication of a single layer of the non-adherent interface covered with chlorhexidine-moistened gauze. When local signs suggestive of BTM infection were observed, dressing changes were performed daily, and the matrix was covered with a neutral non-adherent wound contact layer (CONFORMANT® 2 Wound Veil) and gauze moistened with Colimycin ® reconstituted in normal saline.

Delamination of the sealing membrane was carried out once visual evidence of uniform neodermis maturation covering the entire CBWP area was achieved.

Depending on the case, either a STSG or secondary healing in smaller defects was performed.

In patients undergoing STSG, the graft was covered with a single layer of non-adherent interface (Jelonet ® or equivalent), followed by sterile gauze. The first postoperative dressing change was performed on day 2, then every 2 days, until complete healing. The protocol included gentle cleansing with water and mild soap, and reapplication of a single layer of the non-adherent interface covered with gauze. When local signs suggestive of graft infection were observed, dressing changes were performed daily with application of aqueous chlorhexidine, and the STSG was covered with a neutral non-adherent wound contact layer (CONFORMANT 2 Wound Veil ®) and gauze moistened with Colimycin ® reconstituted in normal saline.

In wounds managed by secondary healing, the neodermis was gently cleansed with water and mild soap at each dressing change and covered with a non-adherent dressing followed by gauze, which was renewed every 2 days, until complete epithelialization. When local signs suggestive of infection were observed, dressing changes were performed daily with application of aqueous chlorhexidine, and Betadine cream ® was applied on the single layer non-adherent interface.

Wound care was predominantly performed on an outpatient basis.

The whole process is schematically represented in Fig. 1.

**Fig. 1:**
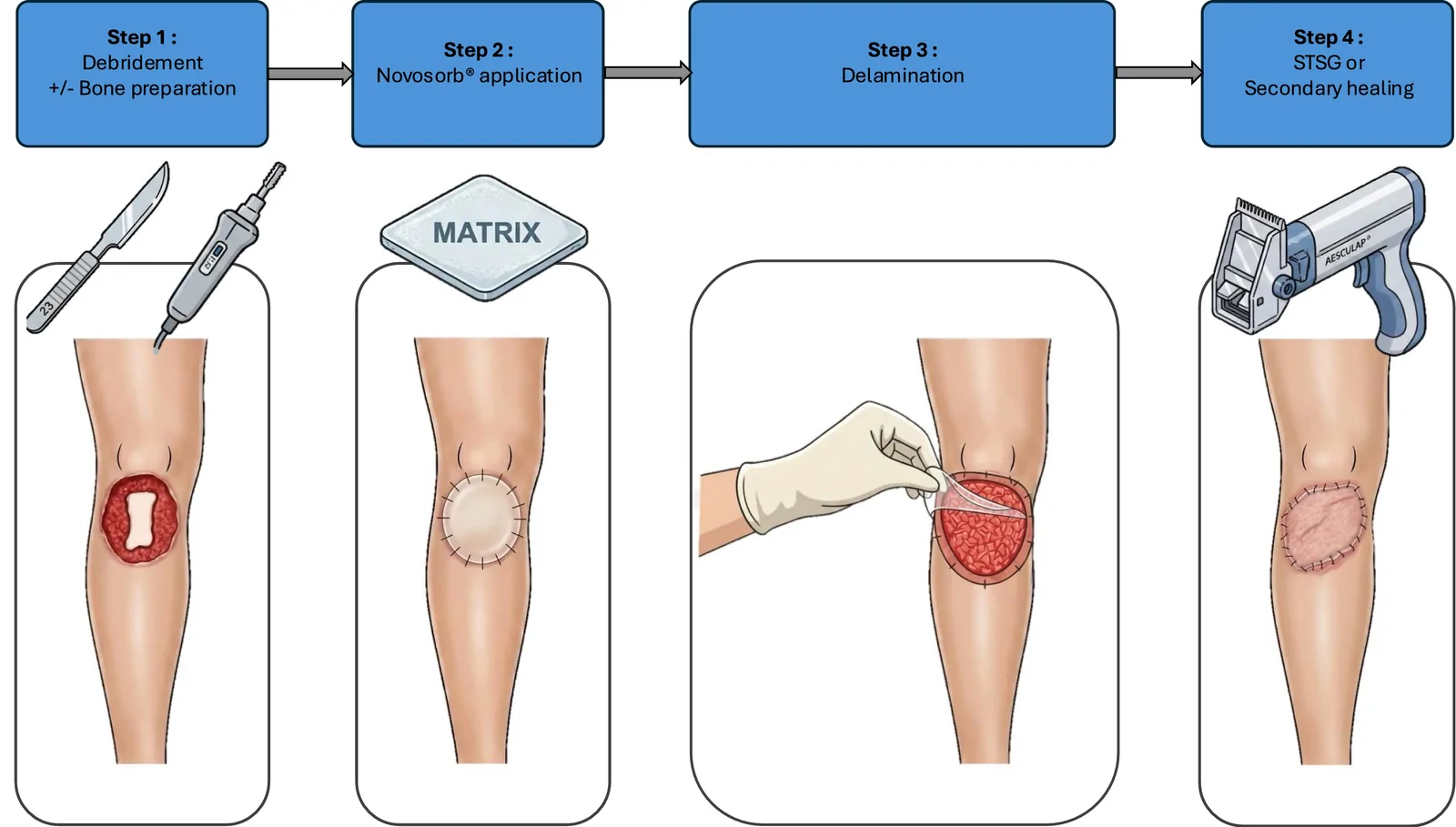
Schematic diagram of the standardized staged reconstruction protocol. After surgical debridement and optional cortical bone preparation, a synthetic dermal matrix is applied over the exposed bone. Following fibrovascular ingrowth and neodermis formation, the sealing membrane is removed (delamination), allowing definitive closure either by STSG (split-thickness skin graft) or secondary healing.

### Follow-up and outcomes

Patients were followed weekly until complete epithelialization to assess both the effectiveness and potential complications of the procedure.

The primary endpoint was the achievement of complete epithelialization, defined as full clinical closure of the wound without further need for dressings.

Secondary endpoints included:

− time to readiness for grafting (days),
− time to complete epithelialization (days),
− complications rate (including infection, hematoma or collection requiring revision; skin graft lysis over the neodermis when performed; and protocol failure needing conversion to an alternative reconstructive method), separating minor complications (not requiring matrix removal) from major complications (requiring matrix removal and change of reconstructive strategy).

### Data collection and statistical analysis

Demographic data, medical history, comorbidities, wound characteristics, operative details, and outcomes were recorded.

Continuous variables were expressed as mean ± standard deviation or median [IQR] depending on distribution. Categorical variables were presented as n (%). Time-to-event outcomes were presented with Kaplan-Meier survival curves. Group comparisons were performed using Mann-Whitney or Kruskal-Wallis tests for continuous variables, and Chi-square or Fisher’s exact tests for categorical variables.

A two-sided α level of 0.05 was considered statistically significant.

Statistical analyses were performed using Jamovi, and GraphPad Prism software.

### Ethics

The study followed the Declaration of Helsinki, and all the patients gave their informed consent. This retrospective series evaluated outcomes of a standard-of-care dermal matrix routinely used in our department; no experimental interventions were performed.

## Results

### Patient characteristics

Between November 2024 and October 2025, 19 consecutive patients presenting with 22 defects exposing CBWP were included.

The mean age was 68.4 ± 13.3 years, and 13 were male patients. Main comorbidities included anticoagulant or antiplatelet drugs in 7 cases, and active smoking in 2 cases.

Defects were located on the scalp (n = 14), tibia (n = 5), forehead (n = 2), and chest wall (n = 1). Mean defect size after initial surgery and before BTM application was 41.5 ± 36.1 cm^2^. Etiologies included oncologic resection (n = 17), burn (n = 4), and trauma (n = 1). Fig. 2, Fig. 3 illustrate representative clinical cases.

**Fig. 2:**
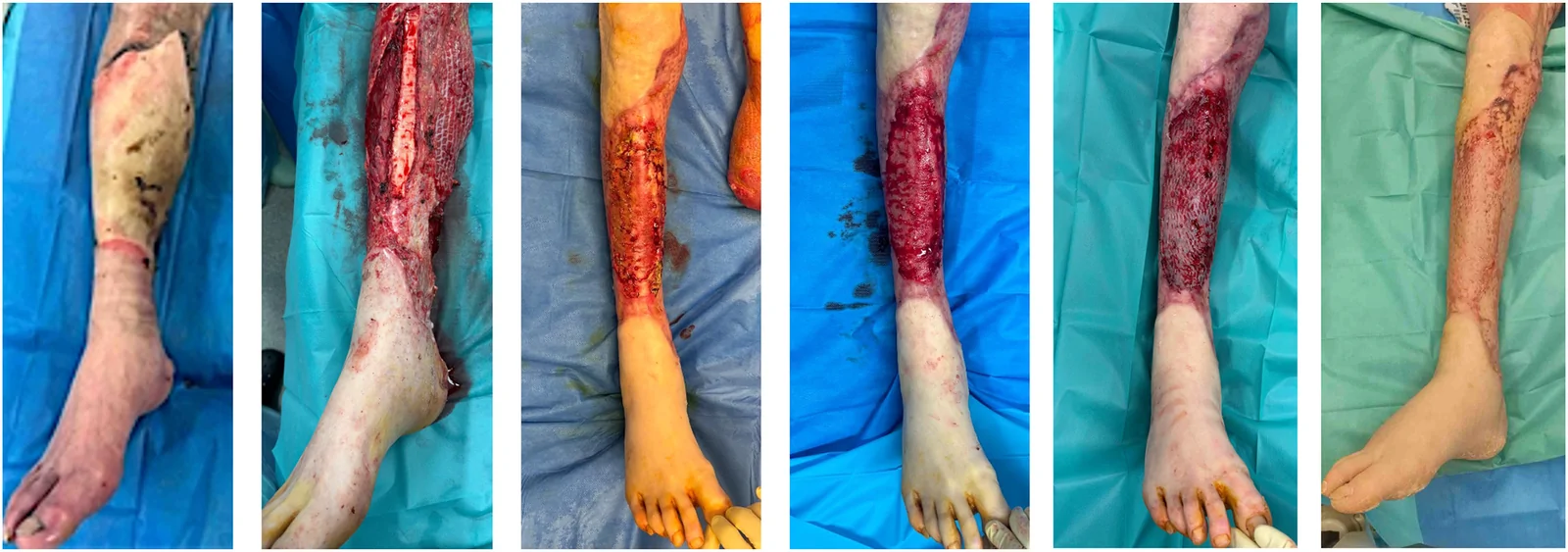
Clinical case, tibia defect, burn. 44-year-old male with 3rd degree burns over 10% total body surface area leading to CBWP over right tibia after an attempt of simple excision and skin graft. The defect measured 20 × 7 cm, with a modified defect size of 3,5 cm. After cortical burring under general anesthesia, matrix was applied, and definitive closure was achieved by split-thickness skin grafting at day 33 with a complete epithelialialization at day 49.

**Fig. 3:**
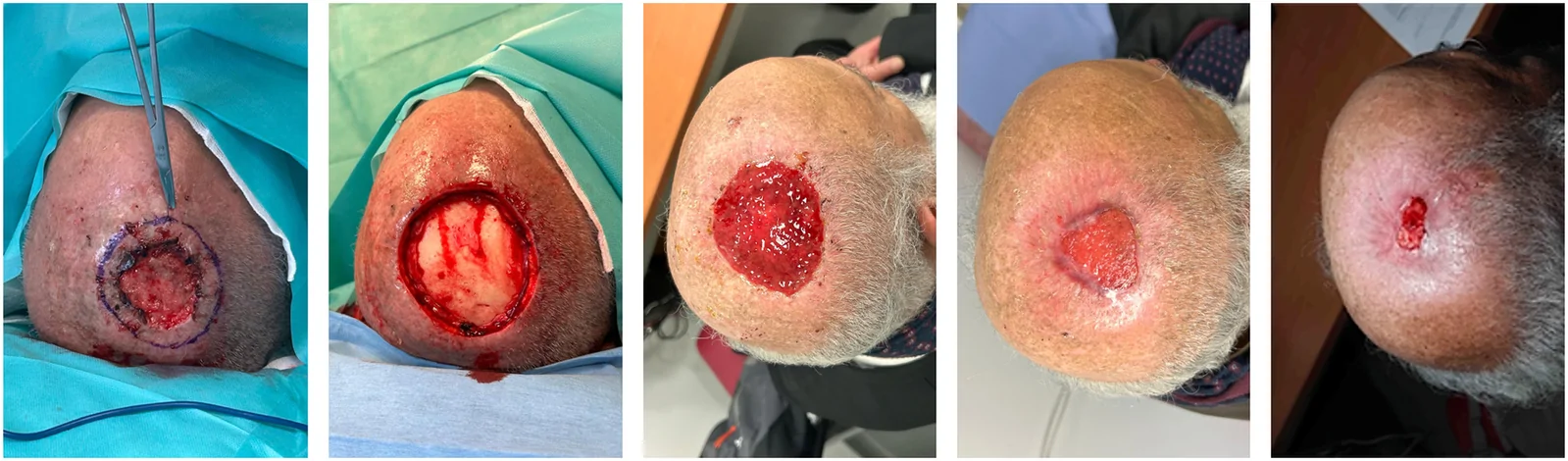
Clinical case, scalp defect, BCC. 70-year-old male with BCC over scalp, extending deep to galea. After excision surgery under local anesthesia, with lateral margins and deep margins including periosteum, the defect measured 7 × 7 cm with a modified defect size of 3.5 cm. Matrix was applied, leading to neodormis formation achieved at day 38, and secondary healing was performed with a complete epithelialization at day 75.

At presentation, 2 defects demonstrated local colonization or infection, controlled prior to matrix application through targeted antibiotic therapy.

Among oncologic cases, 3 patients required neoadjuvant or adjuvant therapy, eg sonidegib or anti-PD1 therapy and 7 were planned for postoperative radiotherapy. No patients were receiving active chemotherapy at the time of matrix implantation.

The initial indication for surgery was tumor re-excision in 9 cases, primary tumor excision in 6 cases, wound debridement in 6 cases, and scar revision in 1 case.

In all cases (22/22), the indication for matrix application was exposure of CBWP.

Baseline, wounds characteristics and surgical indications are summarized in Table 1.

**Table 1:** Baseline, wound characteristics and surgical indications.

Variable		All cases, n = 22
Age, in years	Mean (SD)	68.4 (13.3)
Sex	n (%)	
	Male	15 (68.2%)
	Female	7 (31.8%)
Smoking status	n (%)	
	Non smoker	20 (90.9%)
	Smoker	2 (9.1%)
Anti-coagualants or AAP drugs	n (%)	
	No	15 (68.2%)
	Yes	7 (31.8%)
BMI, in kg/m^2^	Mean (SD)	25 (3.9)

Evolution time, in months	Mean (SD)	10.8 (10.3)
Depth, including	n (%)	
	Hypodermis	1 (4.5%)
	Galea	3 (13.6%)
	Periosteum	18 (81.8%)
Location	n (%)	
	Scalp	14 (63.6%)
	Tibia	5 (22.7%)
	Forehead	2 (9.1%)
	Thorax	1 (4.5%)
Etiology	n (%)	
	Basal cell carcinoma	6 (27.3%)
	Squamous cell carcinoma	8 (36.4%)
	Trichilemnal tumor	2 (9.1%)
	Darier Ferrand tumor	1 (4.5%)
	Burn	4 (18.2%)
	Traumatic necrosis	1 (4.5%)
Wound infection	n (%)	
	No	20 (90.9%)
	Yes	2 (9.1%)
Adjuvant therapy	n (%)	
	No	19 (86.4%)
	Yes	3 (13.6%)
Radiation therapy	n (%)	
	No	15 (68.2%)
	Yes	7 (31.8%)
Surgical indication	n (%)	
	Tumor excision	6 (27.3%)
	Tumor re-excision	9 (40.9%)
	Wound debridment	6 (27.3%)
	Scarring process	1 (4.5%)
Indication for BTM application	n (%)	
	Bone without periosteum	22 (100%)
Defect size (after initial surgery), in cm2	Mean (SD)	41.5 (36.1)

SD = Standard deviation.

### Procedure

Most cases were performed in an outpatient setting (12/22), while 8 patients required conventional hospitalization and 2 patients were admitted to the intensive care unit due to overall clinical condition in the context of severe burns.

BTM application was performed under local anesthesia in 12 cases and general anesthesia in 10 cases.

Regarding bed preparation, BTM was applied after excision, re-excision or debridement without bone preparation in 17 cases, and with bone preparation (bone drilling and/or microperforation) in 5 cases. Fig. 4 represents the links between surgical procedure, anesthesia performed, etiology and wound location.

**Fig. 4:**
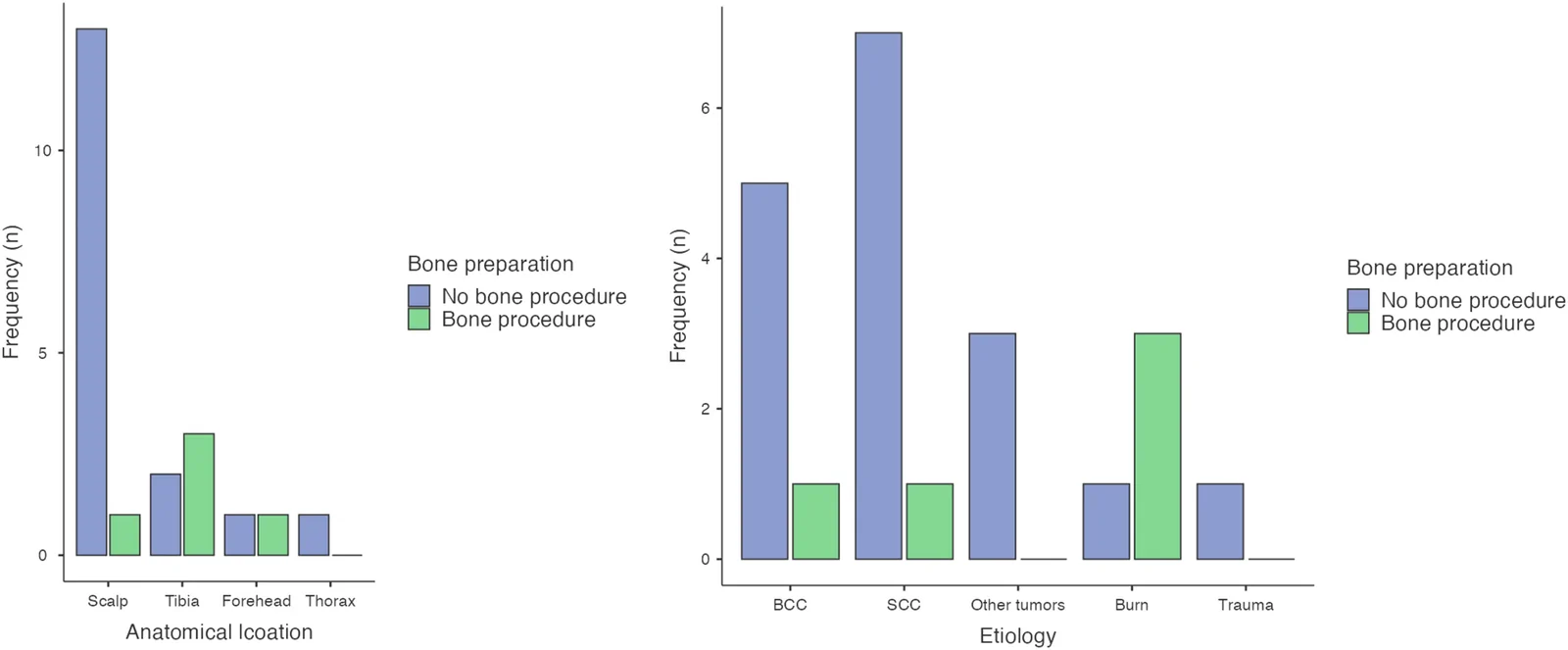
Relationship between surgical procedure, anatomical location and wound etiology (from left to right). BCC = basal cell carcinoma. SCC = Squamous cell carcinoma. Other tumors = Trichilemmal tumor and Darier Ferrand sarcoma.

Procedural characteristics are summarized in Table 2.

**Table 2:** Procedural characteristics.

Variable		All cases, n = 22
Type of hospitalization	n (%)	
	Outpatient	12 (54.5%)
	Conventional stay	8 (36.4%)
	Reanimation stay	2 (9.1%)
Anesthesia	n (%)	
	Local	12 (54.5%)
	General	10 (45.5%)
Surgery	n (%)	
	Excision + Novosorb	6 (27.3%)
	Re-excision + Novosorb	9 (40.9%)
	Debridment + Novosorb	2 (9.1%)
	Bone drilling + Novosorb	1 (4.5%)
	Debridment + Bone drilling + Novosorb	2 (9.1%)
	Debridment + Bone drilling + Microperforations + Novosorb	2 (9.1%)
BTM application size, in cm2	Mean (SD)	41.5 (36.1)

Operative variables of the staged protocol.

BTM = Biodegradable temporizing matrix.

SD = Standard deviation.

### Overall clinical outcomes

One case was excluded from healing analysis due to early discontinuation following positive deep oncologic margins. A flow chart diagram is proposed in Fig. 5.

**Fig. 5:**
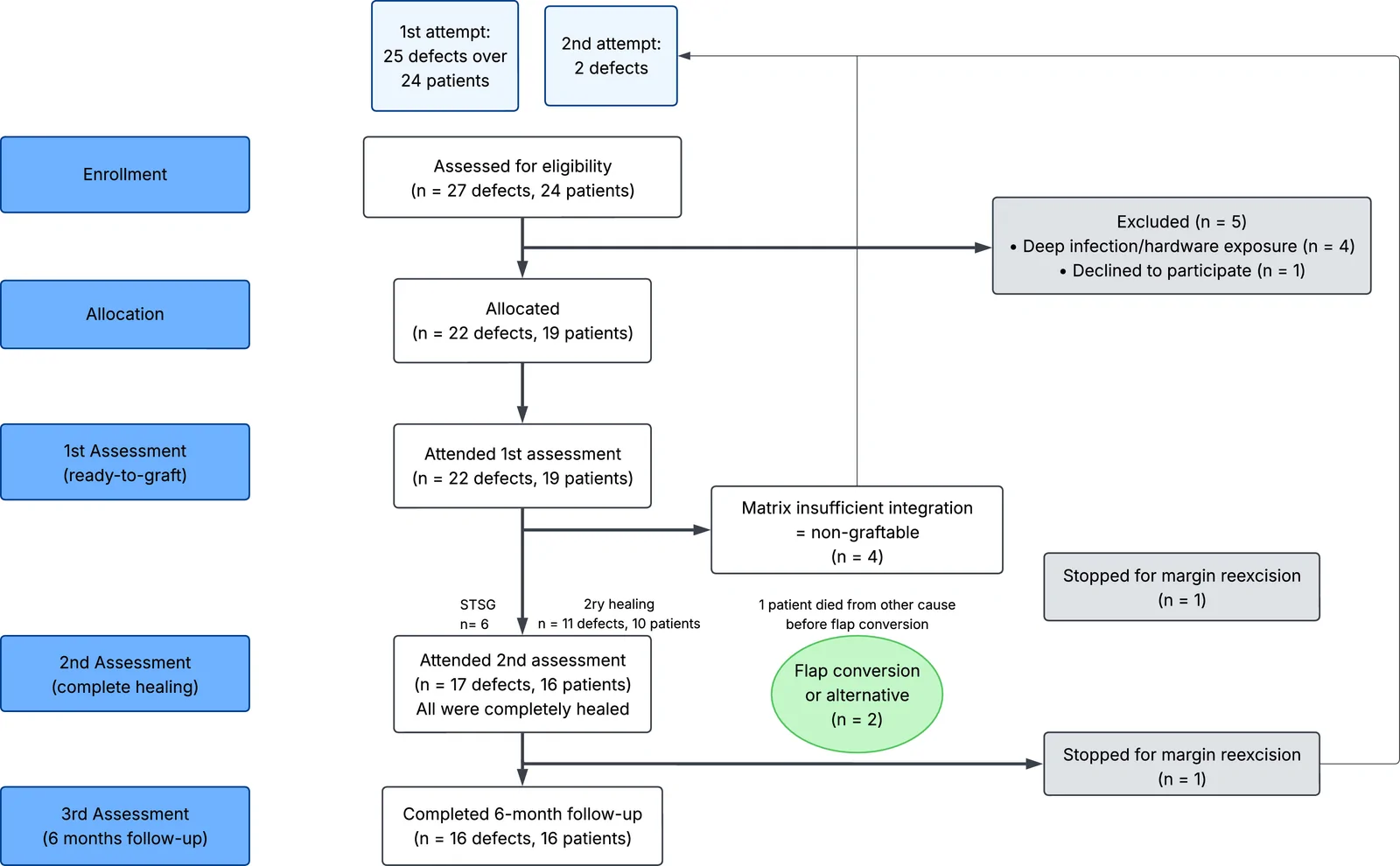
Study flowchart. STSG = Split-thickness skin graft.

Among the remaining 21 defects, successful complete epithelialization without flap reconstruction was achieved in 17 cases.

Median time to readiness for grafting was 37.5 (IQR 12) days.

Among the ready-to-graft defects, 7 underwent STSG after delamination, while 11 were let to heal by secondary epithelialization. STSG procedure is shown according to wound size in Fig. 6.

**Fig. 6:**
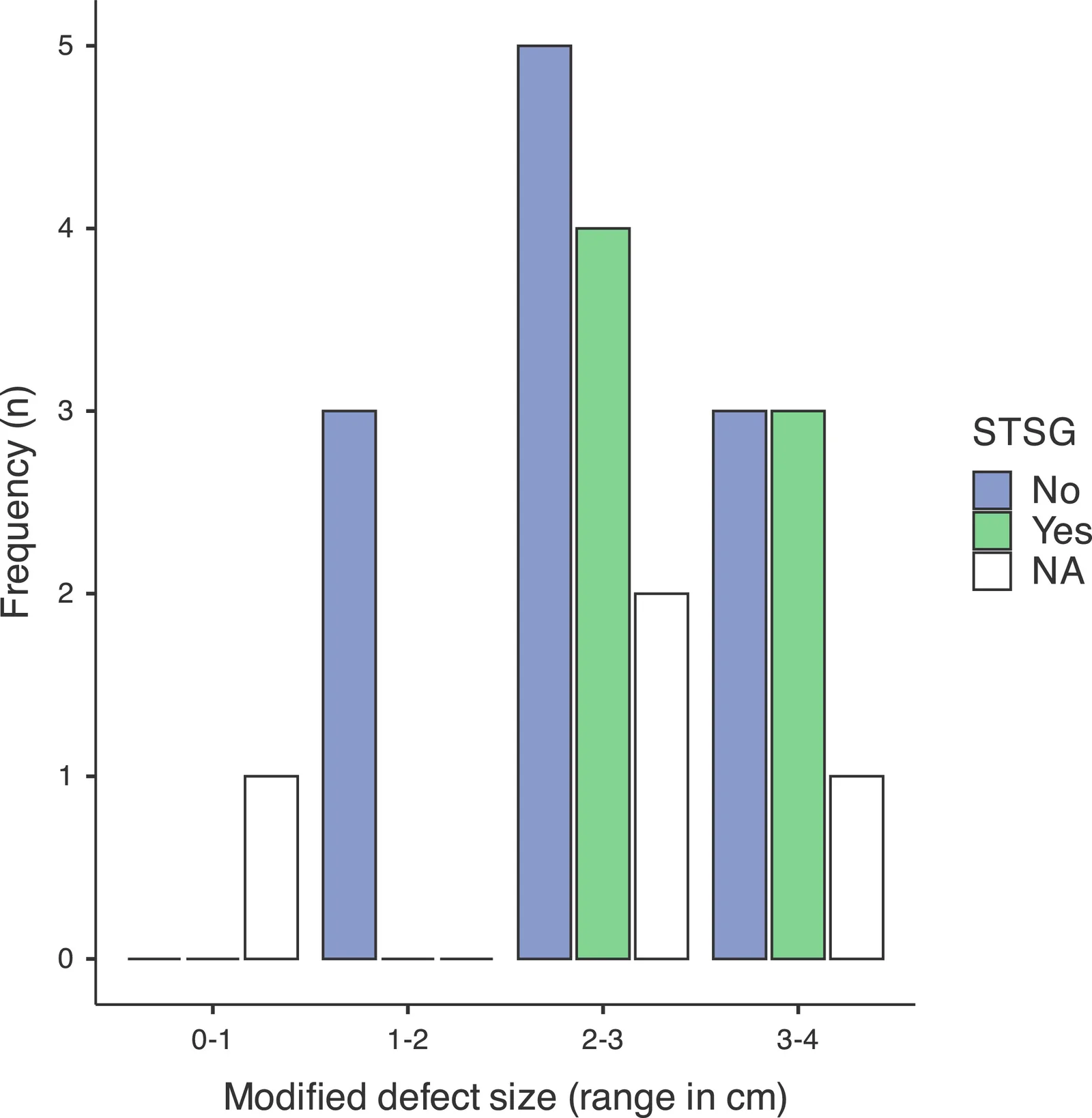
Use of split-thickness skin graft according to modified defect size. Distribution of second step reconstruction (STSG or 2ry healing). Modified defect size is defined in the article. Defect sizes are grouped in ranges of 1 cm. STSG = Split-thickness skin graft. NA = Not applicable, concerns the cases with insufficient matrix integration or stopped process.

Median time to complete epithelialization was 65 (IQR 24) days, with 55 days in the STSG group compared to 71 days in the secondary epithelialization group (p = 0.05)

Results are summarized in Table 3. Time-to-event curves are illustrated in Fig. 7.

**Table 3:** Clinical outcomes and complications.

Variable		All cases, n = 22
Scarring obtained	n (%)	
	Yes	17 (77.3%), 17/21^a^ (81%)
	No	4 (18.2%) 4/21^a^ (19%)
	Not applicable^a^	1 (4.5%)
Graftable, at day	Median (IQR)	37.5 (12)^b^
Grafted	n (%)	
	Yes	7 (38.9%)^b^
	No	11 (61.1%)^b^
Graft take, in % of graft	Mean (SD)	89 (10)
Complete scarring, at day	Median (IQR)	65 (24)^c^
	Minimum - Maximum	34 - 229
Complications, all types	n (%)	6 (27.3%)
Major complications	n (%)	4 (18.2%)
Minor complications	n (%)	2 (9.1%)

a 1 Case excluded from final analysis, refer to Flow chart.

b n = 18, median was used because of variable distribution.

c n = 17, median was used because of variable distribution SD = Standard deviation. IQR = Interquartile range.

**Fig. 7:**
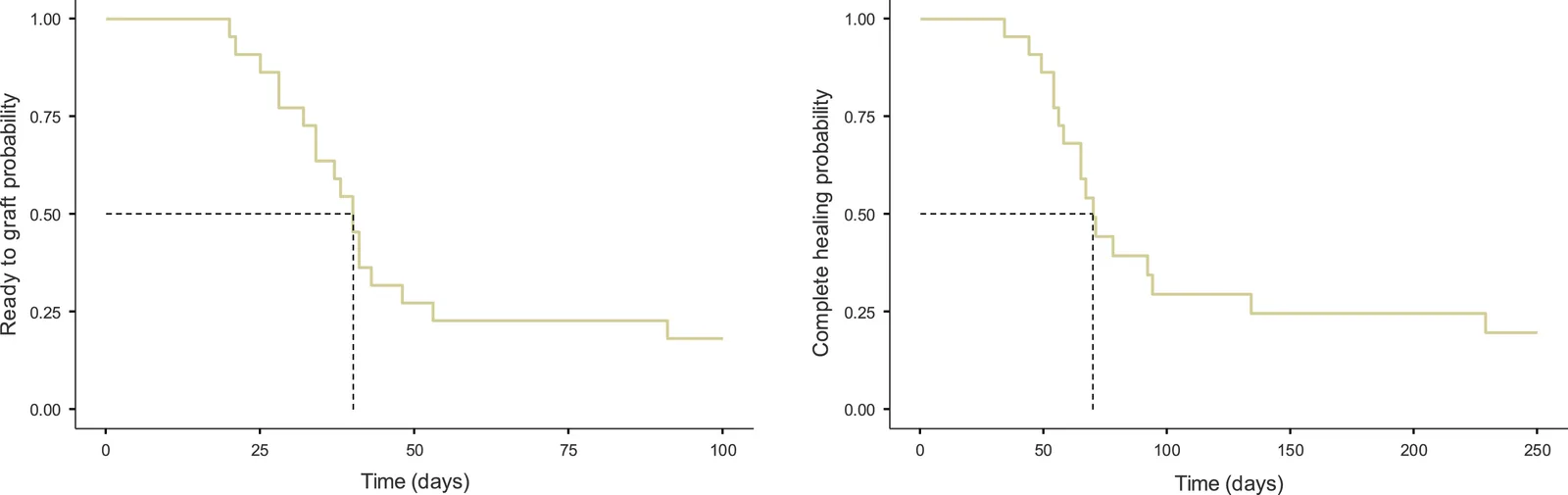
Time-to-event analysis for graft readiness and complete healing. Kaplan-Meier curves illustrating time to readiness for grafting (on the left) and time to complete healing (on the right) following application of the synthetic dermal matrix.

### Complications

At postoperative follow-up, we identified 6 cases with at least 1 postoperative complication. We divided these into major and minor complications (major complication rate, n = 4; minor complication rate, n = 2). Major complications, defined as ones requiring surgical treatment – thus including failure with conversion to another technique -, reanimation, or long-term functional sequalae comprised: 3 insufficient matrix integration requiring conversion to an alternative reconstruction strategy, and 1 hematoma on postoperative day 1 requiring evacuation and matrix replacement. No cases of systemic sepsis or procedure-related mortality were observed. Minor complications, defined as ones treated with simple cares – thus including partial loss -, or drugs only comprised: 2 infections managed conservatively with systemic antibiotics and local dressing protocol without matrix removal.

The overall flap conversion rate was 2/22. One patient died from another systemic cause during the process.

Complications are summarized in Table 3.

### Subgroup analyses

Univariate analyses were performed, evaluating etiology, anatomical location, defect size, bone preparation procedure, and use of STSG. It showed most importantly:

− Comparable healing rates and ready-to-graft time between groups with different etiologies, anatomical location, defect size, and bone preparation (p > 0.05).
− A significant shortened time for complete healing with STSG (55 vs 71 days, log rank test p = 0.015). Curves are shown in Fig. 8.

**Fig. 8:**
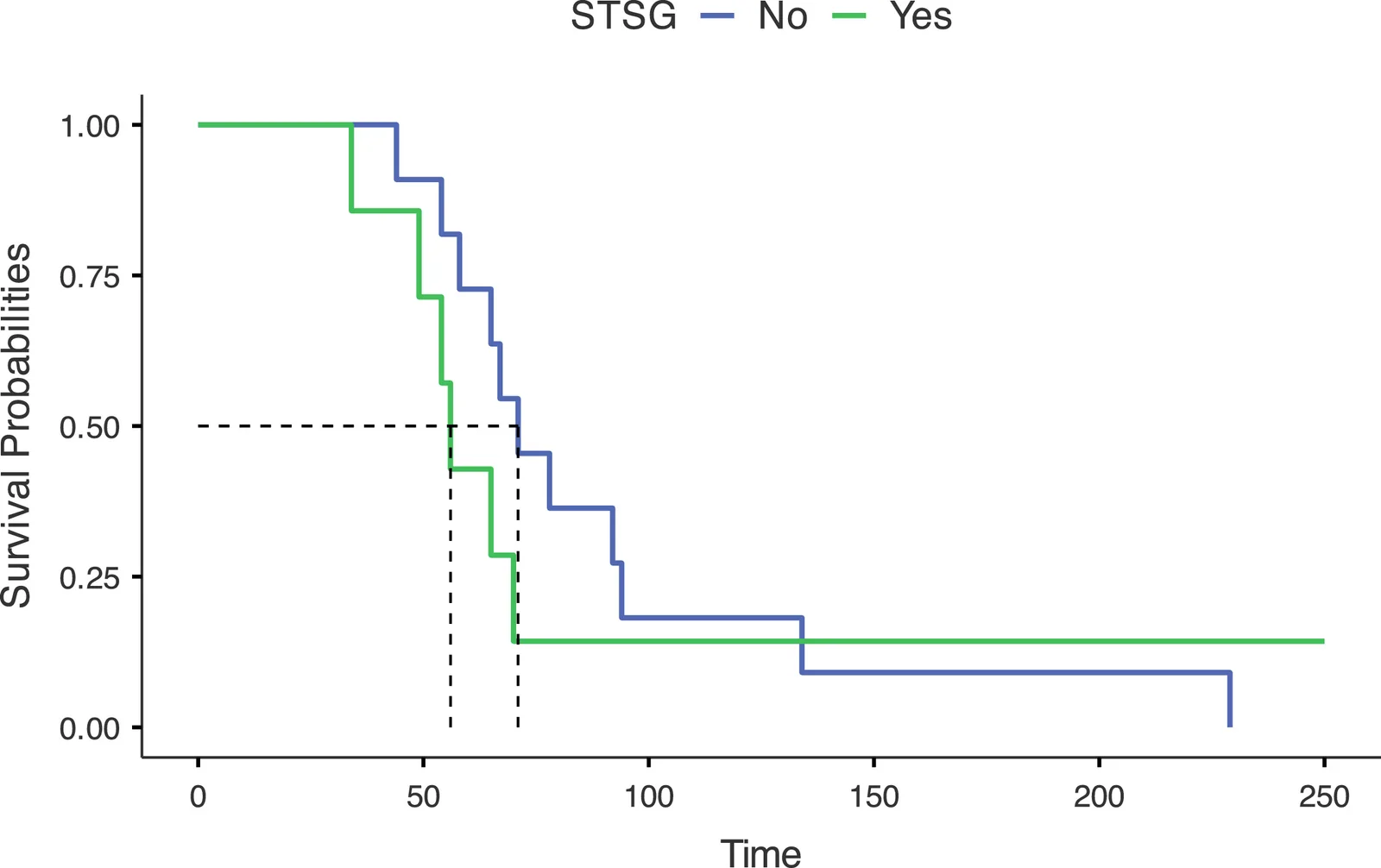
Time to complete healing according to reconstruction strategy. Kaplan-Meier curves comparing time to complete epithelialization between wounds treated with split-thickness skin grafting and those managed by secondary healing.
− A moderate correlation between modified defect size and ready-to-graft time (r = 0.52, p = 0.02).

### Long-term follow-up

At 6-month follow-up, all successfully treated wounds remained stable without recurrence of bone exposure.

No significant scar contracture or late matrix-related complications were observed. Patients were satisfied with functional and aesthetics results.

## Discussion

Soft-tissue defects exposing CBWP represents a common challenging and burdensome coverage problem in plastic surgery. In the absence of periosteum, the cortical surface remains relatively avascular, limiting spontaneous granulation, precluding STSG and prolonging care if no radical treatment is performed.^1^^,^^2^^,^^16^

Traditional strategies rely on cortical burring or drilling to elicit bleeding and waiting for slow granulation, sometimes combined with NPWT.^7^^,^^8^ Although these techniques may eventually generate granulation tissue, the process remains temporally unpredictable and may require prolonged wound care. Alternatively, flap reconstruction provides definitive coverage but entails longer operative time, hospitalization, and donor-site morbidity—considerations that may be disproportionate in elderly, oncologic, or medically frail patients.^9^, ^10^, ^11^

Other strategies have been explored as the formation of an induced-membrane using PMMA cement to replace the periosteum and then allow skin-grafting.^23^

In the present series, a staged synthetic dermal matrix protocol achieved complete epithelialization without flap reconstruction in 81% of defects, across multiple anatomic sites, with favorable and reliable timelines associated with limited morbidity.

Median time to readiness for grafting was 37 days, and median time to complete epithelialization was 65 days. We identified a correlation between healing time and modified defect size. The modified defect size was calculated as the maximal linear between opposing wound edges, representing the theoretical distance required for complete granulation assuming uniform centripetal granulation from the wound margins. Moreover, we didn’t establish a precise size cut-off value, but we assume that STSG should be considered in defects with a large modified defect size, as it shortens the overall time to complete epithelialization.

No clinical or procedural variables were significantly associated with the protocol failure and unsuccessful matrix integration, although the sample size limits definitive conclusion.

Overall, our results reflect the relative homogeneity of the protocol and indication.

Physiologically, the periosteum provides the principal vascular contribution to cortical bone. Once removed, the exposed cortex does not bleed unless perforated, limiting intrinsic angiogenic potential.^1^

Our findings support the concept that a synthetic dermal matrix can bridge avascular beds. NovoSorb® BTM, a fully synthetic biodegradable polyurethane matrix, acts as a porous scaffold that facilitates fibrovascular ingrowth from wound edges and any exposed cortical if bone preparation is performed. The external sealing membrane protects the wound from contamination and mechanical disruption during neodermis formation, prior to STSG or secondary healing. This mechanism is represented in Fig. 9.

**Fig. 9:**
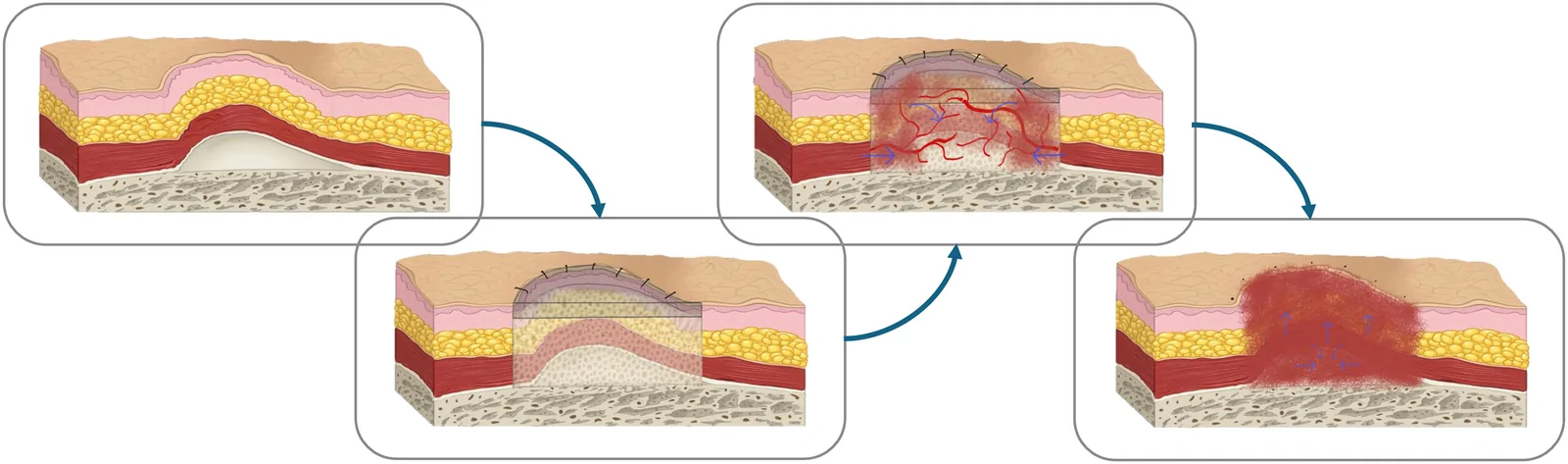
Mechanism of neodermis formation over cortical bone without periosteum. Conceptual illustration showing fibrovascular ingrowth through the dermal matrix from the periphery. Progressive neodermis formation enables skin grafting or secondary healing.

Based on this rationale, we introduced the concept of modified defect size that we defined previously and found consistent in our analysis. As a matter, bone preparation could be particularly relevant only for large modified size defects.

For example, a tibial defect may be a long wound but narrow in width, resulting in a relatively small modified defect size and therefore a shorter effective healing distance considering the bridge mechanism from edges.

Once granulation is obtained, modified defect size should again influence the decision between STSG and secondary healing. Because epithelialization occurs centripetally from the wound margins, larger modified defect sizes are expected to prolong total healing time. In such cases, STSG may reduce overall time to closure, despite requiring an additional procedure and approximately 10 to 15 days for graft take and maturation, with usually high integration rates.

The literature on NovoSorb® BTM has expanded rapidly over the past decade. Large series and systematic reviews report integration rate exceeding 80%, with skin graft take rates typically above 85% across heterogeneous wound types, even in the presence of infection or comorbidities. Reported time to readiness for grafting ranges from 30 to 50 days depending on wound complexity. That is consistent with our series findings.

Lane et al. in 2024 analyzed over 1100 cases and reported high integration rates, excellent graft take with acceptable infection profiles.^21^

Grande et al. and Fruergaard et al. similarly demonstrated favorable efficacy and safety across burns, trauma, and oncologic indications.^22^^,^^24^

However, most published cohorts combine various wound etiologies and anatomical sites.

A few focused series support BTM as a flap-sparing option in specific non-graftable complex wounds and in post-oncologic scalp reconstruction, both reporting satisfactory coverage with limited morbidity.^25^^,^^26^

Our series differs in that CBWP was the exclusive indication, with a standardized staged protocol applied.

Importantly, more than half of cases were managed in an outpatient setting under local anesthesia. The technique is operatively simple, as well as the following dressings and short time follow up until prompt complete healing.

However, it requires the use of Novosorb® BTM, which remains relatively costly, and should be weighed against the potential savings associated with flap avoidance and reduce general anesthesia and hospitalization.

Unlike flap reconstruction, which utilizes a finite tissue resource that cannot be reused once harvested, the dermal matrix may be reapplied unlimited times in cases for example of margin re-excision or tumor recurrence, thereby preserving reconstructive flexibility.

The overall complication rate was 27%, with infections occurring in 9% of cases, all managed conservatively without matrix removal. It may delay but not preclude integration. These results are consistent with reported infection rates of 10–15% in the literature.^19^^,^^21^^,^^22^

Maintaining intimate contact between matrix and wound bed, ensuring adequate drainage through fenestrations, and early evacuation of collections appear critical to successful integration. Flap conversion was required in 9% of defects.

Strengths of our study include a homogeneous indication (CBWP only), standardized staged protocol, complete follow-up with no attrition, and time-to-event analysis. Limitations include retrospective design, modest sample size, absence of a direct flap comparison group, and no formal cost-effectiveness analysis. Multivariate analysis should be performed with higher number of cases.

For selected CBWP defects, a staged NovoSorb® BTM protocol can shorten prolonged granulation phases, avoid immediate flap surgery, and standardize timing to definitive closure.

Rather than replacing flap reconstruction, this approach introduces a reliable intermediate step within the reconstructive ladder. It is not a substitute to flap reconstruction in the presence of deep infection or hardware exposure.

The present findings support a pragmatic algorithm shown in Fig. 10.

**Fig. 10:**
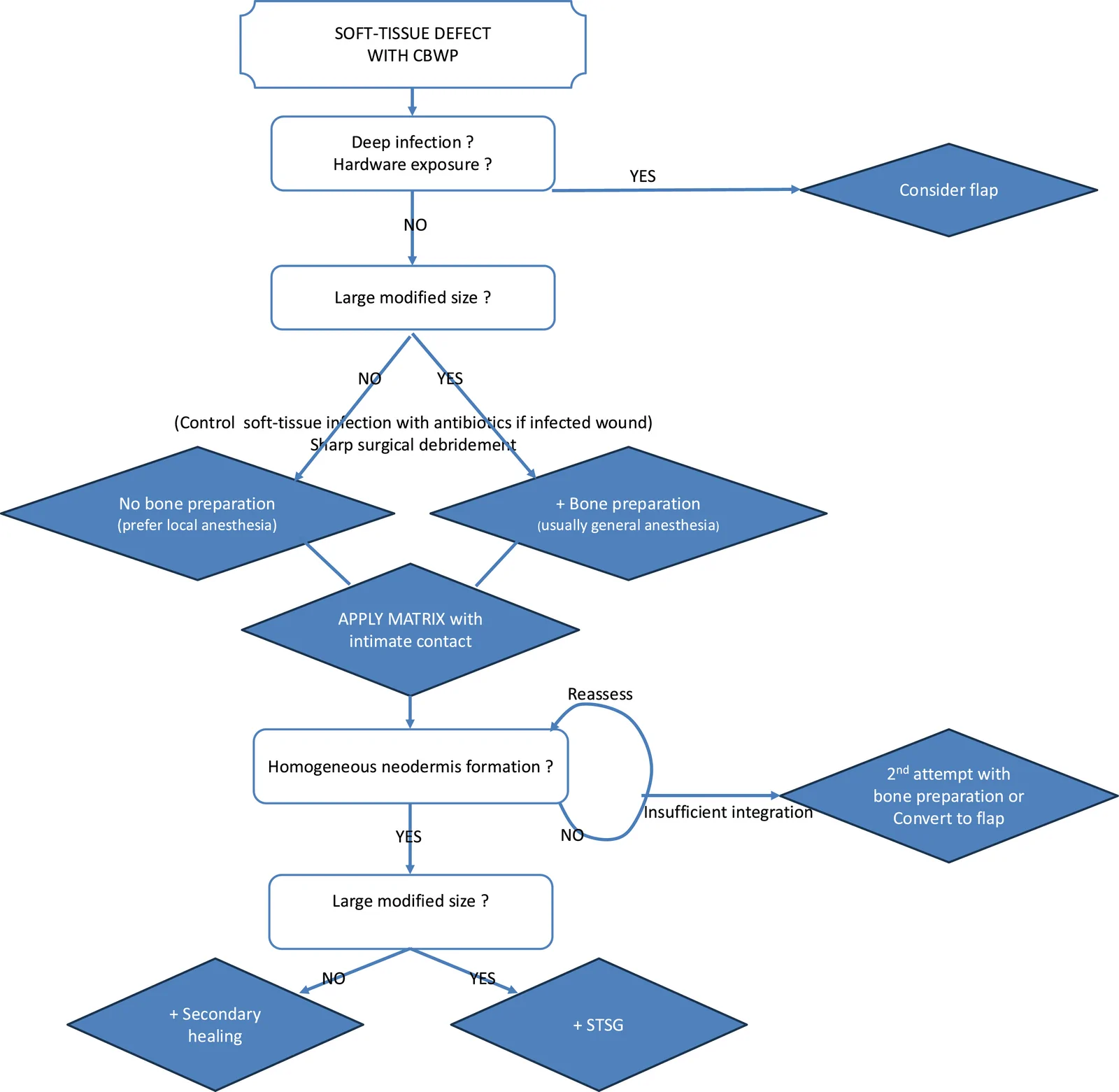
Proposed reconstructive algorithm for defects exposing cortical bone without periosteum. Following thorough surgical debridement, wounds associated with deep infection or hardware exposure are preferentially managed with flap reconstruction. In the absence of these conditions, bone preparation is performed according to the modified defect size, followed by application of the synthetic dermal matrix with intimate contact to the wound bed. Matrix integration is then assessed: successful neodermis formation leads to either split-thickness skin grafting or secondary healing depending on the modified defect size, whereas insufficient integration prompts a second attempt with bone preparation or direct conversion to flap reconstruction.

## Conclusion

Soft-tissue defects exposing cortical bone without periosteum represent a common reconstructive challenge due to the limited vascularity of the cortical surface and the frequent need for flap coverage.

Our results suggest that a simple and safe staged synthetic dermal matrix protocol using NovoSorb® BTM can reliably transform an avascular cortical surface into a vascularized neodermis, enabling definitive closure by skin grafting or secondary epithelialization. In most cases, the procedure can be performed under local anesthesia and in an outpatient setting.

This approach introduces predictability into a traditionally unpredictable healing scenario, with a median time to complete healing of approximately two months.

It represents a valuable intermediate step within the reconstructive ladder for selected defects exposing cortical bone without periosteum, significantly reducing the need for flap surgery.

## Learning points/take home messages

• Cortical bone without periosteum rarely granulates predictably, often requiring complex reconstruction such as flap coverage to achieve definitive wound closure.
• A simple and safe staged synthetic dermal matrix protocol using NovoSorb® BTM can reliably generate a vascularized neodermis over exposed cortical bone, enabling subsequent skin grafting or secondary epithelialization.
• Modified defect size appears more relevant than surface area for predicting healing time, performing bone preparation procedure and choosing between STSG and secondary healing
• By introducing a simple and predictable intermediate step between prolonged granulation and flap reconstruction, BTM may redefine the reconstructive ladder for defects exposing cortical bone without periosteum.

## Authorship statement

All authors have participated to the conception and design, acquisition, analysis, interpretation of data, writing and revision of the article. All authors have approved the final version to be published. There are no other persons who satisfied the criteria for authorship.

## Ethical statement

This study is in accordance with the ethical standards of the Helsinki Declaration (1964) and no approval from an ethic committee was required.

## Patient consent

All the patients gave their informed consent.

## Declaration of competing interest

The authors declare that they have no known competing financial interests or personal relationships that could have appeared to influence the work reported in this paper.
