# Supplementary material for: Biodegradable temporizing matrix in the management of exposed cortical bone without periosteum: a flap-sparing and reproducible strategy
Source: JPRAS Open. 2026 Sep 29;52:603–17. doi: 10.1016/j.jpra.2026.09.031 (PMC13676741; doi:10.1016/j.jpra.2026.09.031)
Supplement: Supplementary file 1 [file mmc1.docx]

**Declaration of interests**
 
☒ The authors declare that they have no known competing financial interests or personal relationships that could have appeared to influence the work reported in this paper.
 
☐ The authors declare the following financial interests/personal relationships which may be considered as potential competing interests:

 
 
 
